# Efficacy and safety of stereotactic radiosurgery for large cystic brain metastases: a comparison with and without cyst aspiration via Ommaya reservoir

**DOI:** 10.1007/s10143-025-03545-7

**Published:** 2025-05-23

**Authors:** Sukwoo Hong, Sheriff Adam, Hirokazu Takami, Motoyuki Umekawa, Yuki Shinya, Hirotaka Hasegawa, Mariko Kawashima, Yousuke Kitagawa, Masashi Nomura, Shunsaku Takayanagi, Nobuhito Saito

**Affiliations:** 1 Department of Neurosurgery, The University of Tokyo Hospital, 7-3-1 Hongo, Bunkyo-ku, Tokyo, 113-8655 Japan; 2 Guy’s, King’s and St Thomas’, King’s College London, London, UK; 3 Department of Neurological Surgery, Mayo Clinic, Rochester, MN USA; 4 Department of Neurosurgery, Saitama Medical Center, Saitama, Japan; 5 Gamma Knife Center, NTT Medical Center Tokyo, Tokyo, Japan; 6 Department of Neurooncology, Saitama Medical University International Medical Center, Saitama, Japan

**Keywords:** Cyst, Aspiration, Ommaya, Hypofractionated, Radiosurgery

## Abstract

To evaluate the therapeutic efficacy and safety of stereotactic radiosurgery (SRS) in the management of large cystic brain metastases. A retrospective analysis was conducted. Large cystic brain metastases were defined as cyst volume ≥ 5 mL. Tumors that received SRS as adjuvant therapy following surgery were excluded. The cohort was stratified into two groups: (1) SRS group (upfront hypofractionated SRS without Ommaya reservoir [OR]), (2) OR-SRS group (cyst aspiration via OR before SRS). Local tumor control rate (LCR), progression-free survival (PFS), overall survival (OS), and radiation-induced adverse events (RAEs) were evaluated. Thirty-one metastases in 28 patients met the inclusion criteria: SRS (14 metastases) and OR-SRS (17 metastases). Most baseline characteristics, including cyst diameter (*P* = 0.15), cyst volume (*P* = 0.06), and tumor volume (*P* = 0.95), were not significantly different between the groups. The cyst volume just before SRS in the OR-SRS group was significantly smaller compared to the SRS group (*P* < 0.01; Mann-Whitney U test). The LCR was 86% in the SRS group and 82% OR-SRS group (*P* = 1.00). Other outcomes, including PFS, RAEs, and OS, were not significantly different between the groups. Kaplan-Meier analysis revealed no statistically significant differences in PFS or OS among the groups (*P* = 0.28 and *P* = 0.20, respectively; log-rank test). This study demonstrates that both SRS and OR-SRS are effective and safe treatment modalities for large cystic brain metastases. The treatment outcomes were comparable, and these approaches may be equally viable options in the management of this challenging subset.

## Clinical trial number

Not applicable.

## Introduction

Brain metastases are the most common type of brain tumors in adults [1, 2]. The main treatment options for local control are surgical resection and radiation, such as stereotactic radiosurgery (SRS) and intraoperative radiation therapy [3–5]. Some brain metastases have a cystic component, and presence of this cystic component is a poor prognostic factor in treatment with surgical resection followed by adjuvant SRS, potentially leading to leptomeningeal dissemination [6]. If the cystic component is large, one treatment option is cyst aspiration before SRS. Cyst aspiration can be performed stereotactically, with or without Ommaya reservoir (OR) placement. The rationale for cyst aspiration before SRS is to reduce the cyst diameter or volume to less than 3 cm or 8 ml, respectively, to avoid radiation-induced adverse events (RAEs) [7, 8]. Furthermore, since the cystic component has been shown to have a lower tumor shrinkage rate [9], cyst aspiration prior to SRS is prudent.

For large brain metastases not amenable to single-session SRS, hypofractionated stereotactic radiosurgery has proven effective [10–12]. With technological advancements in SRS, GammaKnife Icon™ (Elekta Instruments AB, Stockholm, Sweden) has recently made it feasible to perform frameless hypofractionated SRS. While cyst aspiration with or without OR placement has its benefits, it carries the risk of surgical site hemorrhage and wound problems; therefore, some patients would benefit from upfront hypofractionated SRS without preceding cyst aspiration. With the introduction of Icon™ at our facility, our clinical practice has shifted toward upfront hypofractionated SRS. This study aimed to evaluate the treatment outcomes of SRS with or without cyst aspiration for large cystic metastases.

## Methods

This study was approved by the Institutional Review Board (#2231, G10228) of our institution. Written informed consent for participation in the study was obtained from all patients. This study was designed in compliance with the Strengthening the Reporting of Observational Studies in Epidemiology statement [13]. 

### Patient selection and radiological evaluation

**Fig. 1:**
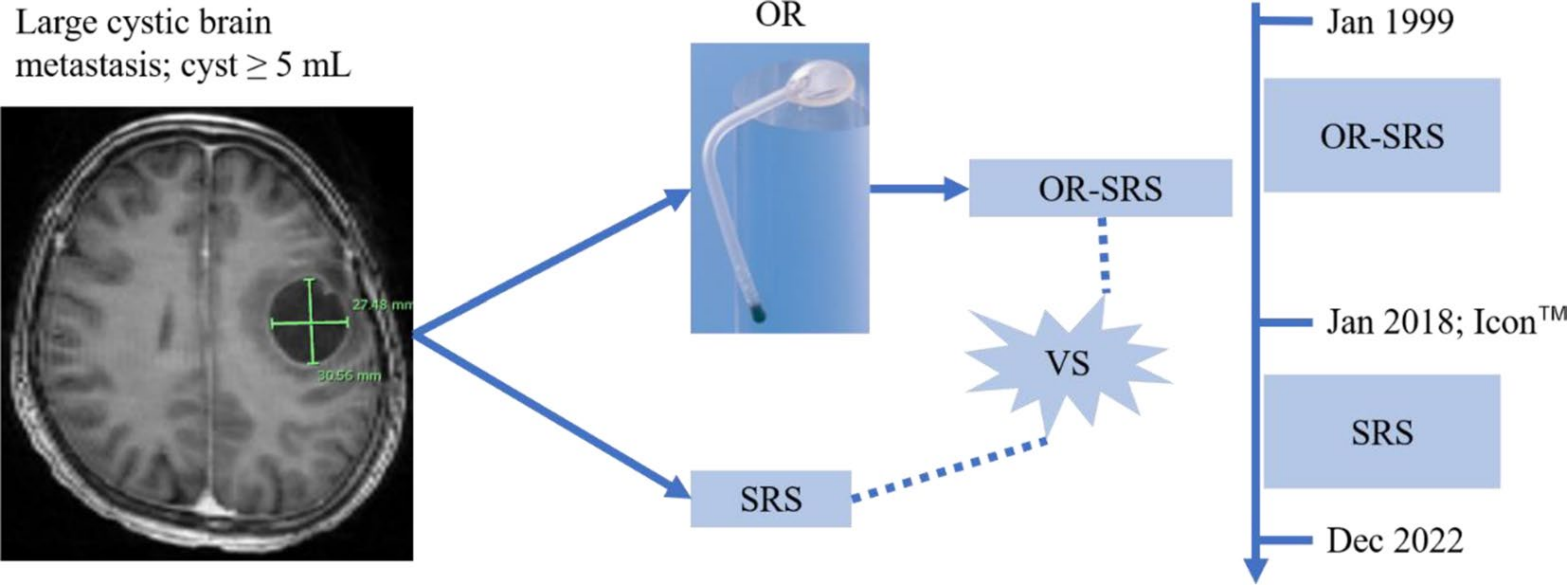
The flowchart illustrates how patients with cystic brain metastases were grouped. Some patients were treated with an Ommaya reservoir (OR) followed by stereotactic radiosurgery (the OR-SRS group) or upfront SRS (the SRS group)

The institutional GammaKnife database was queried for (1) patients treated with OR placement followed by SRS for large cystic brain metastases and (2) patients who were treated with hypofractionated SRS between January 1999 and December 2022 (Fig. 1). Tumors that received SRS as adjuvant therapy following surgery were excluded.

Cystic metastases were defined as a combination of a large cystic portion (cyst volume) and the remaining solid portion (solid volume). Cystic portion was defined as a discrete fluid-filled component hypointense on T1-weighted imaging surrounded by ring-enhancing wall, and iso to hyperintense on T2-weighted imaging [6]. Large cysts were defined as those with cyst volume ≥ 5 mL [14]. Based on diffusion-weighted imaging (DWI), cyst center was labeled as secretion of tumor exudate (low intensity in DWI), and as liquefactive necrosis (iso to high intensity in DWI). Volumes were calculated using Leksell GammaPlan^®^ (Elekta Instruments) based on gadolinium-enhanced magnetic resonance imaging (Gd-MRI). The original tumor volume ($\:=cyst\:volume+solid\:volume$) before OR placement and just before SRS (after aspiration via OR) were calculated.

Fourteen large cystic brain metastases in 12 patients were treated with upfront SRS without preceding aspiration (SRS group). Seventeen large cystic brain metastases in 15 patients were treated with OR placement followed by SRS (OR-SRS group). All ORs were placed using stereotactic guidance. The demographic, radiological, and clinical data of these patients, including graded prognostic assessment (GPA) [15] were collected.

### Radiosurgical procedure

SRS was performed using Leksell GammaKnife (Elekta AB) and all treatment plan were performed using Leksell GammaPlan^®^ (Elekta Instruments). For OR-SRS, after head fixation using a Leksell frame (Elekta Instruments, Stockholm, Sweden), stereotactic MRI was performed to obtain the precise three-dimensional coordinates. Thin-slice Gd-MRI obtained on the day before SRS (the SRS group) and on the day of SRS immediately after cyst aspiration via OR (the OR-SRS group) were co-registered to define the tumor. The median time from OR placement to the SRS was 3 days (interquartile range [IQR] 1.0–6.0 days). All treatments of the SRS group were performed using the GammaKnife Icon™, with sessions divided into either three or five fractions based on each patient’s condition and treatment regimen. Generally, the 3-fraction schedule was completed over three consecutive days, and the 5-fraction schedule over five consecutive days. A thermoplastic mask (Icon Mask Nanor, Elekta) was employed for fixation. A patient marker was placed on the nasal apex to aid in tracking the head movements, which were monitored using infrared light with High-Definition Motion Management. The allowable error margin was 1.5 mm for High-Definition Motion Management.

Typically, a 1-mm tumor margin was outlined with a 50% isodose line, prescribing a standard dose of 20 Gy in single-session SRS, 27–30 Gy in 3 fractions and 30–35 Gy in 5 fractions. For the OR-SRS group, dosing was generally determined based on post-drainage tumor size as follows: if the largest tumor size was < 3 cm, single-session SRS was applied; if the largest tumor size was ≥ 3 cm, SRS was delivered in 3 or 5 fractions based on patient factors. Neither the Ommaya reservoir tract nor the burr hole was intentionally included in the radiation plan. All radiosurgical treatments were planned and approved by dedicated neurosurgeons and radiation oncologists involved in the procedure.

### Follow-up and outcome measurement

The primary outcomes were local tumor control rate (LCR), progression-free survival (PFS), overall survival (OS), and RAEs. Local tumor control was defined as a decrease or stabilization of the treated tumor volume that does not meet the following tumor progression criteria. Tumor progression was defined as ≥ 25% increase in tumor volume [16]. Simple cyst fluid recollection was not regarded as tumor progression. PFS was calculated from the end date of SRS to the date of the following, whichever came first: (1) radiological evidence of tumor progression, (2) the latest date of MRI or enhanced CT if MRI was not feasible, or (3) the date of surgical resection for radiation necrosis developed around the site of SRS. Radiation necrosis was defined as either: 1) < 25% increase in tumor volume or worsening of peritumoral edema that stabilizes over time, with the patient remaining clinically stable, or 2) pathological findings consistent with necrosis without viable cancer cells.

OS was calculated from the end date of SRS to the date of the following: (1) death, or (2) the latest follow-up date. One patient had two metastases which were treated with SRS and OR-SRS. This patient was excluded from the OS comparison between the SRS group and the OR-SRS group. RAEs after SRS procedure were graded per common terminology criteria for adverse events (CTCAE Version 5.0) [17]. 

### Statistical analysis

Given the potential sampling bias, continuous variables were treated as nonparametric and expressed as median values with IQR. Categorical variables were expressed as frequencies and percentages. Chi-square, Fisher’s exact, Mann-Whitney U, and Kruskal-Wallis tests were used as appropriate. Kaplan-Meier curves and the log-rank test were used to evaluate PFS and OS between the groups. Cox regression analysis was performed to evaluate any association of the (pre-)treatment variables with tumor progression and death. Hazard ratios (HRs) with 95% confidence interval (CI) were calculated. The missing data were not imputed. P-values ≤ 0.05 were considered significant. Statistical analyses were performed using SPSS (version 25.0, IBM Corp.).

## Results

### Patient characteristics and treatments

A total of 31 large cystic metastases were identified in 28 patients. The baseline patient characteristics are shown in Table 1. Fourteen metastases (13 patients) were in the SRS group, and 17 (16 patients) were in the OR-SRS group. Two patients had two metastases which were treated differently for each metastasis. Primary cancers included lung cancer (42%), breast cancer (32%), esophageal and rectal cancer (19%), melanoma (3%), and cervical cancer (3%). Of the 15 males, 11 (73%) had lung cancer as the primary cancer. Of the 13 females, eight (62%) had breast cancer as the primary cancer. The proportional difference of lung cancer was significant between men and women (*P* < 0.01). No significant difference was observed in the GPA between the groups (*P* = 0.69).

**Table 1:** Baseline characteristics before treatment

	SRS	OR-SRS	*P*
No. of metastases	14	17	-
No. of pt	13	16	-
Age (IQR), yrs	72 (50–75)	66 (46–75)	0.52
Man sex, n	8 (57%)	7 (41%)	0.38
Neurological symptoms (per pt)	7 (58%)	12 (80%)	0.40
Primary cancer Lung adenocarcinoma Breast Esophagus Rectum Lung SCC Combined lung cancer^a^ Cervix Melanoma	6 (43%) 3 (21%) 2 (14%)D 1 (7%) 0 1 (7%) 0 1 (7%)	5 (29%) 7 (41%) 2 (12%) 1 (6%) 1 (6%) 0 1 (6%) 0	0.48 0.28 1.00 1.00 1.00 0.45 1.00 0.45
Newly diagnosed brain metastasis	13 (93%)	12 (71%)	0.19
GPA (IQR)	1.5 (1.0–2.3)	1.5 (1.0–2.0)	0.69
Largest cyst diameter (IQR), mm	31 (27–35)	35 (29–46)	0.15
Cyst volume (IQR), ml	8.4 (6.5–11.0)	13.7 (7.6–22.2)	0.06
Cyst center Secretion of tumor exudate Central tumor liquefactive necrosis	11 (79%) 3 (21%)	15 (100%)^b^ 0	1.00
Intraoperative fluid removal (IQR), ml	-	13 (8–20)	-
Cyst volume before SRS (IQR), mL	8.4 (6.5–11.0)	2.7 (0.4–3.9)	< 0.01*
Tumor volume (IQR), mL	14.7 (11.5–21.0)	15.2 (9.4–32.7)	0.95

GPA = graded prognostic assessment; IQR = interquartile range; OR = Ommaya reservoir; pt = patient; SCC = squamous cell carcinoma; SRS = stereotactic radiosurgery

^a^ SCLC component 20% and adenocarcinoma component 80%

^b^ Two cyst centers were unknown due to the unavailability of diffusion-weighted imaging

* Statistically significant

**Fig. 2:**
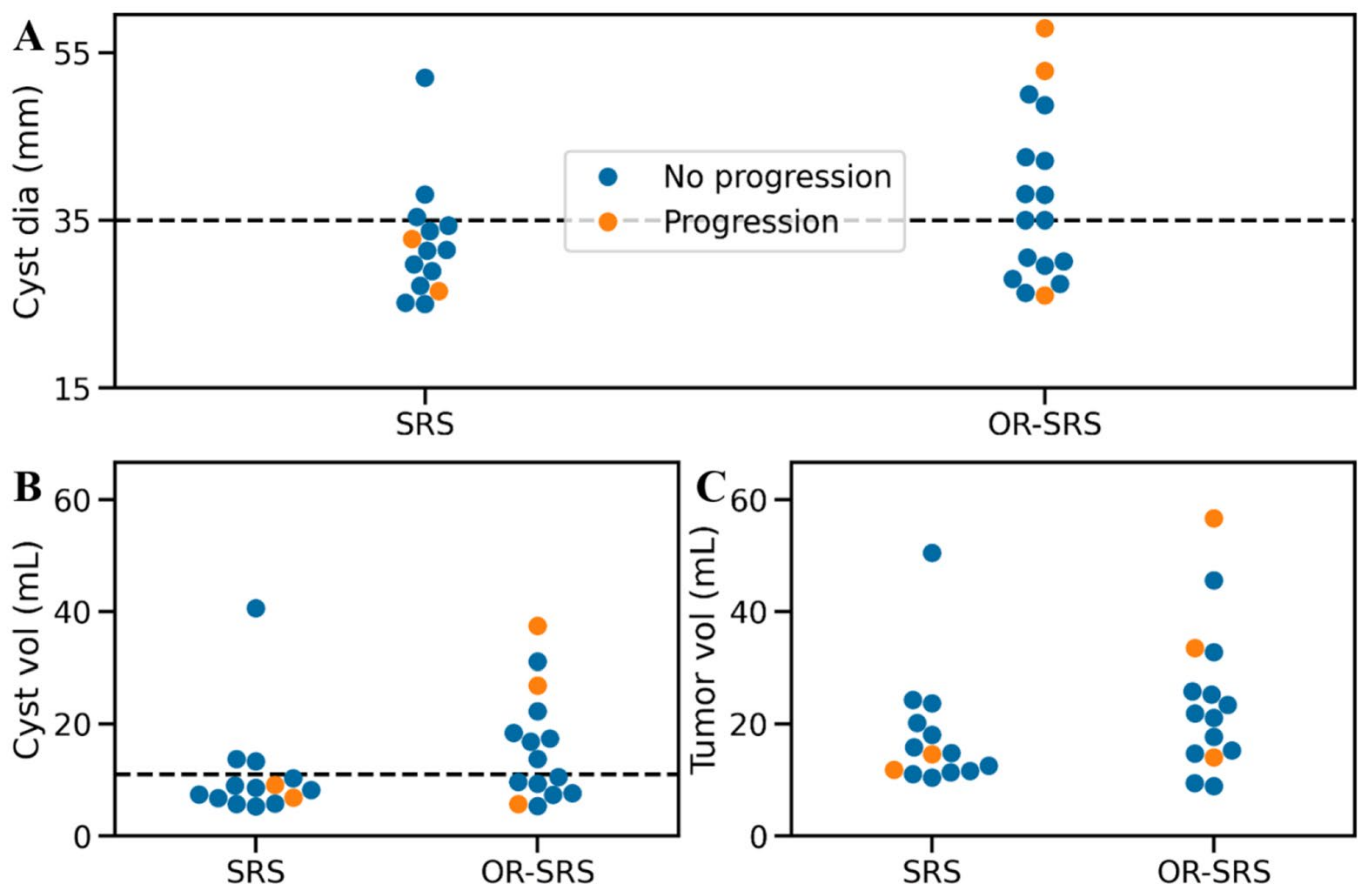
**A**: Swarm plot of the original largest cyst diameter. Dashed line indicates the 75th percentile of the SRS group. **B**: Swarm plot of the original cyst volume. Dashed line indicates the 75th percentile of the SRS group. **C**: Swarm plot of the original tumor volume (cyst + solid)

The median cyst diameter was 35 mm (IQR 29–42 mm). The median cyst volume was 9.3 mL (IQR 7.1–17.1 mL). The cyst volume in the OR-SRS group was not significantly larger than that in the SRS group (*P* = 0.06). The median volume reduction by OR before SRS was 80% (IQR, 69–97%). Swarm plots of the diameters and volumes are shown in Fig. 2.

**Fig. 3:**
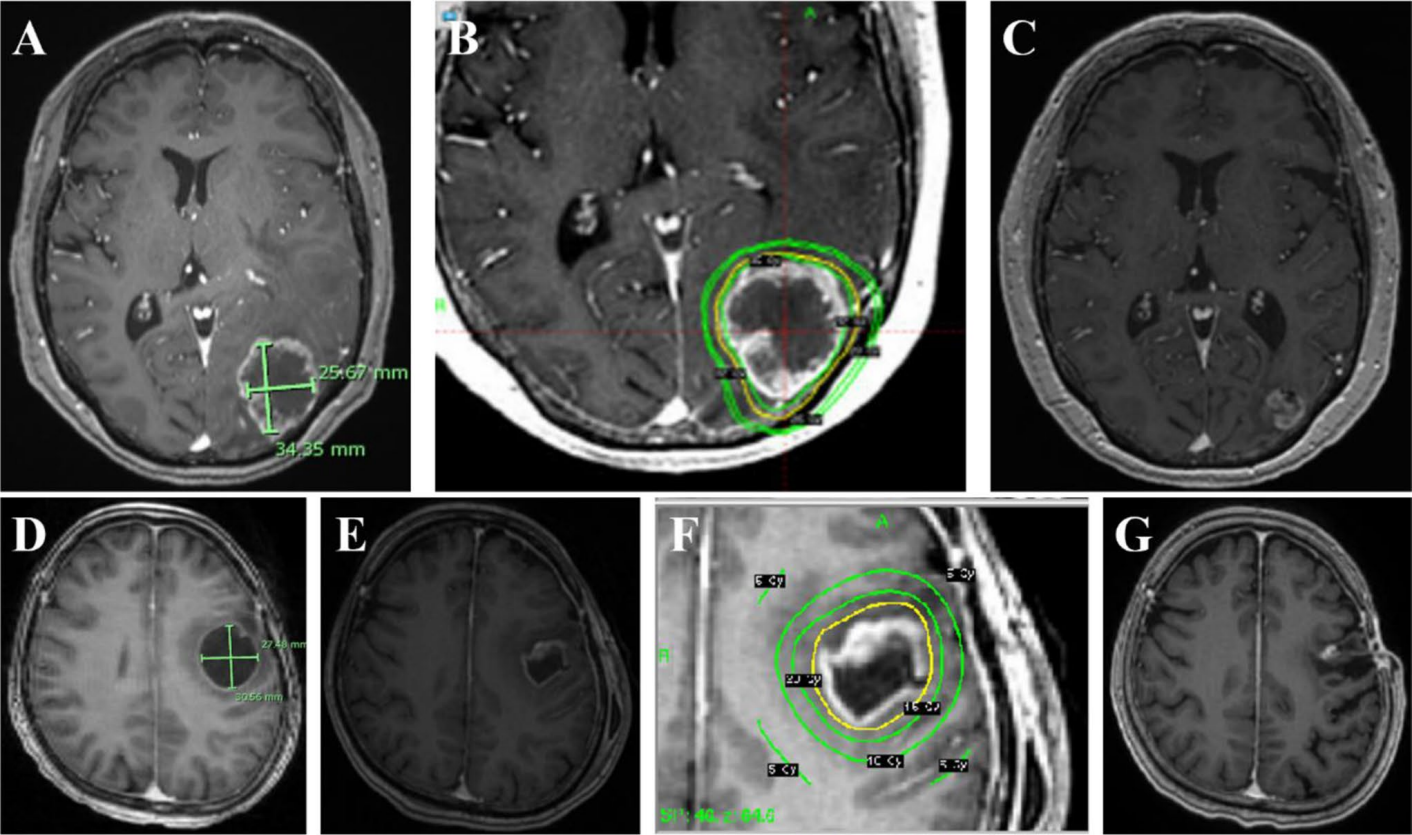
Two illustrative cases in each group. **A**: The initial gadolinium-enhanced magnetic resonance imaging (Gd-MRI) axial view shows ring-enhancing tumor occupying left occipital lobe in 77-year-old woman with lung adenocarcinoma. The longest cyst diameter was 34.35 mm, and the cyst volume was 13.7 ml. **B**: Hypofractionated stereotactic radiosurgery (SRS) was administered with the marginal dose of 32 Gy over five fractions. **C**: Follow-up imaging in 17 months shows the cystic mass under control. **D**: The initial Gd-MRI axial view shows a ring-enhancing tumor occupying left frontal lobe in 54-year-old woman with breast cancer. The longest cyst diameter was 30.56 mm, and the cyst volume was 10.5 ml. **E**: Ommaya reservoir was placed with cyst aspiration before SRS. **F**: Single-session SRS was administered with the marginal dose of 20 Gy. **G**: Follow-up imaging in 110 months shows the cystic mass under control

SRS parameters are presented in Table 2. In the SRS group, four cystic metastases were treated with 3-fraction SRS, and 10 with 5-fraction SRS. For 3-fraction SRS, the median marginal dose was 30 Gy (IQR 28.5–30 Gy), and the median central dose was 59 Gy (IQR 51.5–63.5 Gy). For 5-fraction SRS, the median marginal dose was 32 Gy (IQR 32–32 Gy), and the median central dose was 64 Gy (IQR 60–66 Gy). In the OR-SRS group, 13 treatments (77%) were single-session, with a median marginal dose of 18 Gy (IQR 17–20 Gy) and a median central dose of 45 Gy (IQR 40–50 Gy). Two illustrative cases are described in Fig. 3.

**Table 2:** SRS parameters

	SRS		OR-SRS			
No. of fractions	3	5	1	2	3	5
No. of metastases	4	10	13	1	2	1
Central dose (IQR), Gy	59 (52–64)	64 (60–66)	45 (40–50)	45^a^	50, 54^a^	60^a^
Marginal dose (IQR), Gy	30 (29–30)	32 (32–32)	18 (17–20)	18^a^	27^a^	32^a^

IQR = interquartile range; OR = Ommaya reservoir; SRS = stereotactic radiosurgery.

^a^ No IQR because the total number is small.

### PFS, OS, and associated risk

The outcomes of SRS are shown in Table 3. The median radiological follow-up period was 203 days (IQR 51–600 days).

**Table 3:** SRS outcomes

	SRS	OR-SRS	*P*
LCR, n	12 (86%)	14 (82%)	1.00
Progression, n	2 (14%)	3 (18%)	1.00
PFS (IQR), days	133 (9–352)	148 (55–553)	0.44
RAE	2 (14%)	4 (24%)	0.66
CNS necrosis	2 (14%)	2 (12%)	1.00
Wound problem	0	1 (6%)	1.00
Death, n	10 (83%)	9 (60%)	0.24
OS (IQR), days	334 (101–701)	285 (124–1821)	0.58

CNS = central nervous system; HF = hypofractionated; IQR = interquartile range; LCR = local tumor control rate; OR = Ommaya reservoir; RAE = radiation-induced adverse event; SRS = stereotactic radiosurgery

**Fig. 4:**
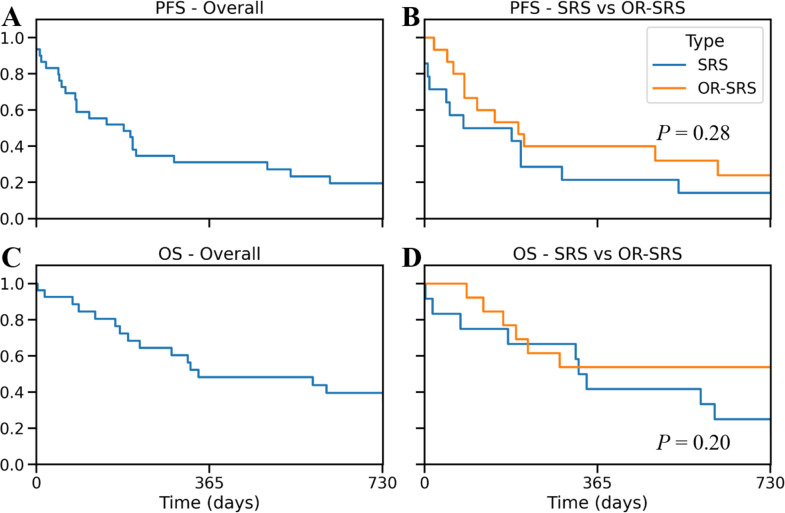
**A**: Progression-free survival (PFS) of all the patients. **B**: PFS was not significantly different between the groups. **C**: Overall survival (OS) of all the patients. **D**: OS was not significantly different between the groups

The overall LCR was 84%, with no significant differences between the groups (*P* = 1.00). One progression (6%) in the OR-SRS group was near the Ommaya reservoir tract. Kaplan-Meier curves for PFS and OS are shown in Fig. 4.

There were no statistically significant differences in LCR (*P* = 0.28) and OS (*P* = 0.20) between the groups. Univariate Cox regression analysis for tumor progression did not show any significant variables (Table 4).

**Table 4:** Univariate Cox regression analysis for progression

	HR (95% CI)	*P*
Age (continuous)	1.04 (0.96–1.13)	0.33
Man	7.72 (0.85–7.02 × 10)	0.07
Primary cancer Lung adenocarcinoma Breast Esophagus Rectum Lung SCC Combined lung cancer ^a^ Cervix	Ref 1.00 (0.09–1.11 × 10) 1.00 (0.07–1.35 × 10) 1.00 (0.00–1.12 × 10^4^) 1.00 (0.01–1.55 × 10^2^) 1.00 (0.03–3.82 × 10) 1.00 (0.03–3.82 × 10)	1.00 1.00 1.00 1.00 1.00 1.00
Newly diagnosed brain metastasis	26.38 (0.00–1.45 × 10^6^)	0.56
GPA (continuous)	0.64 (0.22–1.88)	0.64
Largest cyst diameter (continuous)	1.03 (0.92–1.16)	0.60
Cyst volume (continuous)	1.02 (0.94–1.12)	0.62
Central tumor liquefactive necrosis	0.04 (0.00–9.22 × 10^2^)	0.52
Tumor volume (continuous)	1.02 (0.95–1.09)	0.57
OR-SRS	1.05 (0.18–6.32)	0.96

GPA = graded prognostic assessment; IQR = interquartile range; OR = Ommaya reservoir; SCC = squamous cell carcinoma; SRS = stereotactic radiosurgery

^a^ SCLC component 20% and adenocarcinoma component 80%

**Fig. 5:**
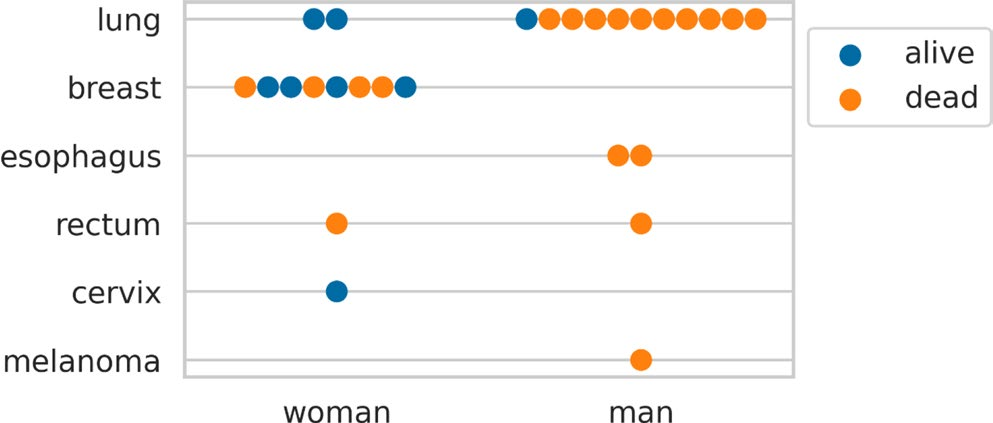
Alive or deceased status at the latest follow-up, categorized by sex and primary cancer origin. Note that more than half of the survivors have cancer originating from female-specific organs

Univariate Cox regression analysis for death (Table 5) showed that man sex was a significant factor (HR 5.49, 95% CI 1.55–19.4, *P* < 0.01). GPA, cyst diameter or volume, and treatment group were not significant factors for death. At the latest follow-up, of the eight survivors, seven were women, and five of them had primary cancers originating from the breast or cervix (Fig. 5).

**Table 5:** Univariate Cox regression analysis for death

	HR (95% CI)	*P*
Age (continuous)	1.02 (0.98–1.05)	0.37
Man	5.49 (1.55–1.94 × 10)	< 0.01*
Primary cancer origin Lung Breast Esophagus Rectum Cervix Melanoma	Ref 1.00 (0.34–2.92) 1.00 (0.21–4.89) 1.00 (0.02–4.71 × 10) 1.00 (0.17–5.87) 1.00 (0.00–3.26 × 10^4^)	1.00 1.00 1.00 1.00 1.00
Newly diagnosed brain metastasis	0.96 (0.31–2.93)	0.94
GPA (continuous)	0.54 (0.25–1.18)	0.12
Largest cyst diameter (continuous)	1.02 (0.96–1.09)	0.49
Cyst volume (continuous)	0.98 (0.92–1.04)	0.43
Central tumor liquefactive necrosis	0.68 (0.09–5.15)	0.71
Tumor volume (continuous)	0.94 (0.87–1.00)	0.06
OR-SRS	0.54 (0.20–1.42)	0.21

GPA = graded prognostic assessment; OR = Ommaya reservoir; SCC = squamous cell carcinoma; SRS = stereotactic radiosurgery

* Statistically significant

Six RAEs were observed in six patients. In the SRS group, one patient developed grade 1 radiation necrosis, and another experienced grade 1 tumor hemorrhage. In the OR-SRS group, one patient developed grade 4 radiation necrosis requiring surgical intervention four years after SRS, another developed grade 2 radiation necrosis, and a third experienced grade 3 wound dehiscence at the site of the occipital OR placement, necessitating its removal one month after SRS. Tumor hemorrhage (grade 1) was also observed in a different patient. No significant difference was observed in the frequency of RAEs between the groups.

## Discussion

This study summarized the outcomes of two treatment methods involving SRS for large cystic brain metastases. No significant difference was observed in PFS, OS, and the frequency of RAEs between the two treatment methods. The current study suggests that for large cystic brain metastases, the outcomes are comparable between OR-SRS and HF-SRS.

Based on the LCR of 82–86% across the two treatment methods, both forms of SRS can be considered effective for controlling large cystic brain metastases. Each method has its pros and cons. OR-SRS is beneficial in reducing symptoms from mass effect and decreasing cyst volume for subsequent SRS. However, OR-SRS carries risks such as repositioning of the OR tip due to inadequate cyst aspiration (11–22%), surgical site infection (11%), and tumor hemorrhage (11%) [14, 18, 19]. In our OR-SRS group, rates of wound dehiscence (8%) and tumor hemorrhage (8%) were comparable to those reported in previous studies. These adverse events impose additional burdens on cancer patients and should be minimized where possible with upfront SRS. Since one progression was near the Ommaya reservoir tract, including the Ommaya tract—with or without the burr hole—in the radiation plan might have prevented this progression. Although excluded in our cohort, current SRS contouring guidelines for adjuvant therapy to the resection cavity and/or residual tumor recommend including the surgical tract in the radiation plan to prevent progression [20, 21]. Extrapolating this guideline to Ommaya reservoir placement, we could have incorporated the Ommaya tract in the radiation plan.

Upfront SRS has the advantage of reducing invasiveness; however, it is important to note that the size limits for the safe treatment for cystic metastases have not been well studied. Considering the IQR of the largest cyst diameter in the SRS group and the swarm plot (Fig. 2A), cysts up to 35 mm in diameter can be reasonably managed with upfront SRS. Likewise, cyst volume up to 11 mL can be reasonably managed with upfront SRS. For cyst volume larger than 11 mL, it is reasonable to consider OR placement to decrease cyst volume. To the best of our knowledge, no studies have directly compared treatment outcomes between upfront SRS and OR-SRS, despite numerous studies demonstrating the effectiveness of both approaches. The LCR of cystic brain metastases treated with cyst aspiration (with or without OR) followed by SRS has ranged from 68–91% [19, 22–24], which is comparable to the 82% observed in the OR-SRS group of our study. In contrast, studies on upfront SRS have reported LCRs ranging from 93–97% [11, 12], higher than the 86% observed in the SRS group of our study. However, it is important to note that these previous studies broadly included brain metastases and not exclusively large cystic metastases like those in our study, making direct comparisons less appropriate.

Finally, men had a significantly higher HR for death than women; however, this may be due to the proportional difference in lung and breast cancers between the sexes. The similar result has been reported in the past [25]. The five-year relative survival rate for breast cancer patients with distant metastasis is 31%, which is higher than that for non-small cell lung cancer (9%) and small cell lung cancer (3%) [26, 27]. 

### Limitations

The limitations of the current study include its retrospective nature, small sample size, and heterogeneity of the patient population. The results of our study need to be verified with future prospective or retrospective studies with larger sample sizes, or through a systematic review with meta-analysis of the existing literature.

## Conclusion

Upfront SRS and OR-SRS are both effective and safe treatments for large cystic brain metastases. The treatment outcomes were comparable. Upfront SRS may be a preferable option for patients to avoid surgical risks associated with OR placement. Further studies with larger sample sizes are warranted to validate these findings.

## Data Availability

No datasets were generated or analysed during the current study.
